# Combating respiratory diseases with mucosal vaccines

**DOI:** 10.1128/jvi.00146-26

**Published:** 2026-04-15

**Authors:** Patrick Chun Hean Tang, Zheng Ling, Mabel Lee, Nicholas J. C. King, Suresh Mahalingam

**Affiliations:** 1 Emerging Viruses, Inflammation and Therapeutics Group, Institute for Biomedicine and Glycomics, Griffith University, Gold Coast, Queensland, Australia; 2 Global Virus Network (GVN) Centre of Excellence in Arboviruses, Griffith University, Gold Coast, Queensland, Australia; 3 School of Pharmacy and Medical Sciences, Griffith University, Gold Coast, Queensland, Australia; 4 Viral Immunopathology Laboratory, Infection, Immunity and Inflammation Research Theme, Charles Perkins Centre, School of Medical Sciences, Faculty of Medicine and Health, The University of Sydney 4334, Sydney, NSW, Australia; 5 Sydney Institute for Infectious Diseases, The University of Sydney 4334, Sydney, NSW, Australia; Universiteit Gent, Merelbeke, Belgium

**Keywords:** vaccines, mucosal vaccines, mucosal immunity, respiratory viruses, immunization, mRNA, live vector vaccines

## Abstract

Mucosal vaccines hold many advantages over parenteral vaccines in the prevention of respiratory infections, which remain a major global health burden. Unlike parenteral vaccines, which primarily induce systemic immunity, mucosal vaccines stimulate robust local immune responses, including secretory IgA, tissue-resident memory T and B cells, and mucosal IgG. These responses collectively block viral entry, limit replication, and reduce transmission. Critically, they provide protection at the site of infection, offering rapid and durable immunity. This review highlights recent advances in mucosal vaccine platforms and delivery strategies for respiratory viruses, including emerging vaccine platforms, adjuvants, and heterologous prime-boost approaches, with a focus on findings from both animal models and human studies that define the key immune components and mechanisms of action in mucosal immunity. We further discuss evidence across major respiratory viruses and the translational and clinical implications for next-generation vaccine design. These insights underscore the role of mucosal vaccines in strengthening future pandemic preparedness.

## INTRODUCTION

Respiratory diseases are a major public health concern worldwide, causing significant negative consequences for global health and economic growth. In 2021, the World Health Organization (WHO) reported lower respiratory tract (LRT) infections as the fifth leading cause of death, claiming 2.5 million lives globally (1). Severe LRT viral infections are frequently caused by influenza virus, respiratory syncytial virus (RSV), and human metapneumovirus (hMPV) in both the elderly and the young. Alarmingly, however, in this year, the novel rapidly emerging coronavirus, COVID-19, overtook this group of LRT infections to become the second leading cause of death globally, in itself claiming 8.8 million lives. COVID-19 can infect the upper respiratory tract (URT), LRT, or both, with the worst outcomes generally associated with LRT infections. Within the WHO European region, the International Respiratory Coalition reported 219,500 deaths in 2021 from LRT infections, costing up to €109 billion (2), while 1.6 million deaths from COVID-19 cost €829 billion (3). The importance of vaccination was highlighted by a 2017 study showing that, while vaccination may not dramatically reduce total hospitalization costs (€1,184,808 vs €1,152,333), it substantially reduces disease severity, complications, and years of life lost (4); this was further supported by a 2022 Turkish influenza study reporting an almost fivefold higher mean patient hospitalization cost in unvaccinated than vaccinated patients, related to the need for intensive care (5).

The 1918 influenza pandemic (H1N1) marked the worst pandemic in recorded history, with approximately 50 million deaths worldwide (6). Two subsequent influenza pandemics, the Asian Flu (H2N2) in 1957 and the Hong Kong Flu (H3N2) in 1968, each caused about 1 million deaths (7). In 2009, another influenza pandemic caused by a novel H1N1 virus claimed over 250,000 lives (8). Genomic and phylogenetic analyses showed that the virus arose through multiple reassortment events in swine, incorporating gene segments from classical swine H1N1, North American triple-reassortant, and Eurasian avian-like swine influenza lineages (9). The capacity of influenza virus to reassort gene segments makes novel influenza pandemics an ever-present threat.

RSV, another leading cause of LRT infections, was estimated to have caused 3.6 million pediatric hospitalizations and 101,400 deaths in children under 5 years old globally in 2019, with approximately 50% of RSV-related deaths occurring in infants under 6 months old (10, 11). RSV also infects elderly adults, with 159,000 hospitalizations per year in adults aged over 65 years in the USA (12). In RSV-infected adults aged 50–59 years, the annual economic burden was estimated at $6.6 billion, including $1.1 billion in hospitalization costs (13).

Although hMPV is less prevalent than influenza and RSV, hMPV causes severe LRT infections. A 2009–2014 study in Germany found hMPV in 131 of 4,776 pediatric respiratory cases (2.7%), with 65% requiring hospitalization (14). In New Zealand (2012–2015), 276 of 6,427 adult hospitalizations (4.3%) were linked to hMPV (15).

While endemic coronavirus infection is a relatively common seasonal URT infection causing mild cold symptoms in many communities, in 2003, an epidemic outbreak in Guangdong Province, China, caused by a novel coronavirus, SARS-CoV, caused severe respiratory symptoms and spread quickly. The potential pandemic was narrowly averted when public health control measures brought it under control, bringing coronaviruses to world attention (16). In 2012, a MERS-CoV outbreak subsided due to inefficient virus transmission between humans (17). However, due to a lethal combination of high infectivity and air travel facilitating the rapid seeding of SARS-CoV-2 infection hotspots worldwide, as well as delays in public health responses, the 2019 COVID-19 pandemic became inevitable. In an accelerated response to this pandemic, peripherally administered mRNA vaccines against COVID-19, developed on existing platforms, undeniably saved millions of lives. However, adverse side effects, low protection duration, poor cross-protection against emerging variants of concern, continued virus transmission in vaccinated individuals, and their storage and transportation requirements have emerged as significant limitations (18, 19). Most importantly, while the majority of SARS-CoV-2 vaccines administered parenterally induce effective systemic immunity, they do not induce local mucosal immunity, i.e*.*, at the site of infection (20, 21). In light of this, multiple organizations have pushed for the development of a second-generation COVID-19 vaccine program (22). Recently, CEPI announced a $57 million project to develop inhaled and nasal vaccines against SARS-CoV-2 and variants of concern (23).

## MUCOSAL IMMUNITY AGAINST RESPIRATORY VIRUSES

### Overview

Medically important respiratory viruses, like human coronaviruses (HCoVs), influenza viruses, RSV, and hMPV, are highly transmissible pathogens that overcome innate mucosal barriers to infect the underlying tissues (24–26), particularly airway epithelium (27–29). Infection may remain in the URT (i.e.*,* nose, sinuses, pharynx, larynx, and trachea), causing coryzal symptoms due to inflammation and swelling of the mucosa, and/or infect the LRT, involving bronchi, bronchioles, and alveoli, where involvement of the smaller airways and alveoli may compromise air exchange. Irrespective of the site, infection triggers a programmed set of similar sequential, intercalated responses between the innate and adaptive immune systems that serve to control and eradicate infection and generate robust local and systemic immunity against reinfection (Table 1).

**TABLE 1:** Key differences between mucosal and systemic immunity on primary exposure

	Adaptive immunity	
	Mucosal immunity	Systemic immunity
Immune function	First line of defense to prevent respiratory virus entry—mucus reduces contact with epithelium	Clearance of respiratory viral infection if respiratory mucosal barrier is breached
Anatomical locations	Mucosal surfaces and MALTs in respiratory tract	Blood, internal organs, and lymphatic system, including spleen and lymph nodes
Key antibodies	Non-specific S-IgA (dimeric)	Serum IgM, IgG, monomeric IgA
Key memory lymphocytes	T_rm_ and B_rm_ cells with emergence of adaptive response	Circulating memory T and B cells

Initial detection of, and responses to, inhaled pathogens usually occur in the URT and likely determine disease severity and outcome. Mucosa-associated lymphoid tissues (MALT) of the URT, i.e., the nasopharyngeal-associated lymphoid tissues (NALT) in mice, and the palatine and nasopharyngeal tonsils in humans, are an important site for first pathogen contact. They enable early interaction between crucial *in situ* cellular components of the innate and adaptive immune response, obviating the delay incurred by migration of these cells to lymph nodes required at other barrier sites like the skin to initiate these responses. Throughout the respiratory tract, interspersed within follicle-associated epithelium (FAE) that covers the MALT collections are microfold (M) cells, epithelial cells specialized to capture luminal antigens and invading microorganisms. For example, influenza viruses are transcytosed to antigen-presenting cells (APCs) on the basal side of the FAE (30), facilitating efficient lymphocyte priming and recall responses (31–33). Distal inducible bronchus-associated lymphoid tissues (iBALT), which are absent under homeostatic conditions, form *de novo* during influenza virus infection or following SARS-CoV-2 mucosal vaccination (34–36). Induction of long-lasting mucosal CD8^+^ tissue-resident memory T cells (T_rm_) after URT immunization prevents lung reinfection (37). Together, respiratory tract MALTs may act as long-term reservoirs of tissue-resident memory cells (38–40) and represent promising targets for inducing local immunity through mucosal vaccines.

### The innate cellular response—myeloid cells

Macrophages and dendritic cells (DCs) are abundant in the respiratory mucosa, where they phagocytose pathogens and provide immunosurveillance (41–43). During viral infection, myeloid lineage responses can also cause severe immunopathology in the lungs. This arises from their ability to detect pathogens through recognition of pathogen- and damage-associated molecular patterns (PAMPs, DAMPs) via extracellular and intracellular pathogen recognition receptors (PRRs), including Toll-like receptors (TLRs), the retinoic acid-inducible gene I (RIG-I)-like receptors (RLRs), nucleotide-binding oligomerization domain (NOD)-like receptors (NLRs), C-type lectin receptors, and absent in melanoma 2 (AIM2)-like receptors (44) These pathways, which may themselves be strategically manipulated by viruses, have important homeostatic roles but can, during infection, differentially drive increased production of antiviral and proinflammatory mediators. These include type I and III interferons (IFNs) and downstream interferon-stimulated genes, cytokines, such as TNF, IL-6, IL-1, and IL-18 (via TLRs, RLRs, and NLRs), as well as inflammasome activation and PANoptosis (via NLRs and AIM2). Together with increasing IFN-γ and TNF signaling, these responses can trigger a cytokine storm, leading to multi-organ failure and death.

#### Macrophages

Alveolar macrophages (AMs) patrol the alveoli and intercept pathogens not cleared by proximal physical defenses. They typically degrade external antigens without triggering excessive inflammation under homeostatic conditions (43, 45). AMs help control viral load during influenza and HCoV infection by inducing IFN-independent resistance in epithelial cells and coordinating a controlled proinflammatory response that activates adaptive immunity (46). Depletion of AMs results in severe inflammation, alveolar damage, loss of respiratory function, and mortality (47–50). However, influenza viruses and HCoVs can also infect AMs and survive endosomal and lysosomal degradation, with persistent SARS-CoV-2 infection evading immune detection (51, 52). In SARS-CoV-2 pneumonia, M1-polarized, rather than M2 AMs, are infected and support viral replication (51, 53), recruiting inflammatory monocytes, T cells, and neutrophils, which cause immunopathology (54–56).

Interstitial macrophages (IMs), phenotypically similar to tissue-resident macrophages in other tissues, reside in the lung parenchyma deep to epithelial cells (43) and are generally more specialized in antigen presentation and induction of inflammatory responses than AMs (45). During influenza and SARS-CoV-2 infection, numbers of IM differentiating from infiltrating pro-inflammatory monocytes rapidly increase and can become pathogenic (57, 58). SARS-CoV-2 infects activated IMs more efficiently than AMs, inducing high levels of inflammatory cytokines and chemokines, including type I IFN, IL-6, CCL2/7/8/13, CXCL10, as well as profibrotic factors, such as transforming growth factor (TGF)-β1 and secreted phosphoprotein 1 (59).

#### Dendritic cells

Dendritic cells (DCs) are broadly classified into plasmacytoid DCs (pDCs) and conventional DCs (cDCs), with the latter subdivided into cDC1 (CD103^+^ in mice; CD141^+^ in humans) and cDC2 (CD11b^+^ in mice; CD1c^+^ in humans) (45, 60). In the respiratory mucosa, cDC1s are positioned near the epithelium, extending transepithelial processes to capture antigen, while cDC2s reside deeper in the interstitium (61–63). After antigen uptake, both migrate to draining lymph nodes, where cDC1s cross-present via major histocompatibility complex (MHC) I to prime CD8^+^ T cells and cDC2s present via MHC II to prime CD4^+^ T cells (64–67). pDCs, located in the lamina propria, are weaker APCs but produce large amounts of type I and III IFNs upon activation (68–70).

Like macrophages, DCs can be infected by respiratory viruses (71). RSV replication in DCs is poor or abortive, yet infected DCs are inhibited from inducing effector T cells through type I and III IFN signaling and RSV nucleoprotein interference, resulting in impaired adaptive immune responses (72–74). During SARS-CoV-2 infection, cDC1 numbers decline, and antigen presentation is reduced, with lower co-stimulatory CD80/CD86 expression, leading to diminished CD4^+^ and CD8^+^ T-cell responses (75–78). Type I IFN production in pDCs is also selectively impaired during SARS-CoV-2 infection, resulting in increased viral replication and tissue damage (76, 79).

#### Innate “memory”—responses to pathogen re-challenge

Macrophages and DCs are pivotal early responders to pathogens, but do not develop antigen-specific memory. Instead, after infection, they can adopt a non-specific hyperresponsive “standby” state that is agnostic to antigen (80) for a significant period. Thus, influenza-infected mice show enhanced protection against *Streptococcus pneumoniae* at 1 month post-infection, mediated by increased IL-6 production from monocyte-derived AMs (81). Similarly, at 30–39 days post-infection with SARS-CoV-2, AMs ameliorate influenza A disease through enhanced type I IFN-related responses (82). Induction of this innate “standby” or “memory-like” state in AMs is thought to depend on CD8^+^ effector T cells and IFN-γ stimulation, pointing to a reciprocal interplay between adaptive and innate cells in immune memory (83). Emerging evidence suggests DCs may also have a similar innate “memory,” exhibiting M1-like phenotypes during fungal lung infection (84). Such memory-like myeloid responses in the respiratory mucosa could confer a substantial advantage for mucosal over peripherally-administered vaccines, considering the statistical likelihood of immediate re-exposure after infection under epidemic conditions.

### The adaptive cellular response—lymphocytes

**Fig 1:**
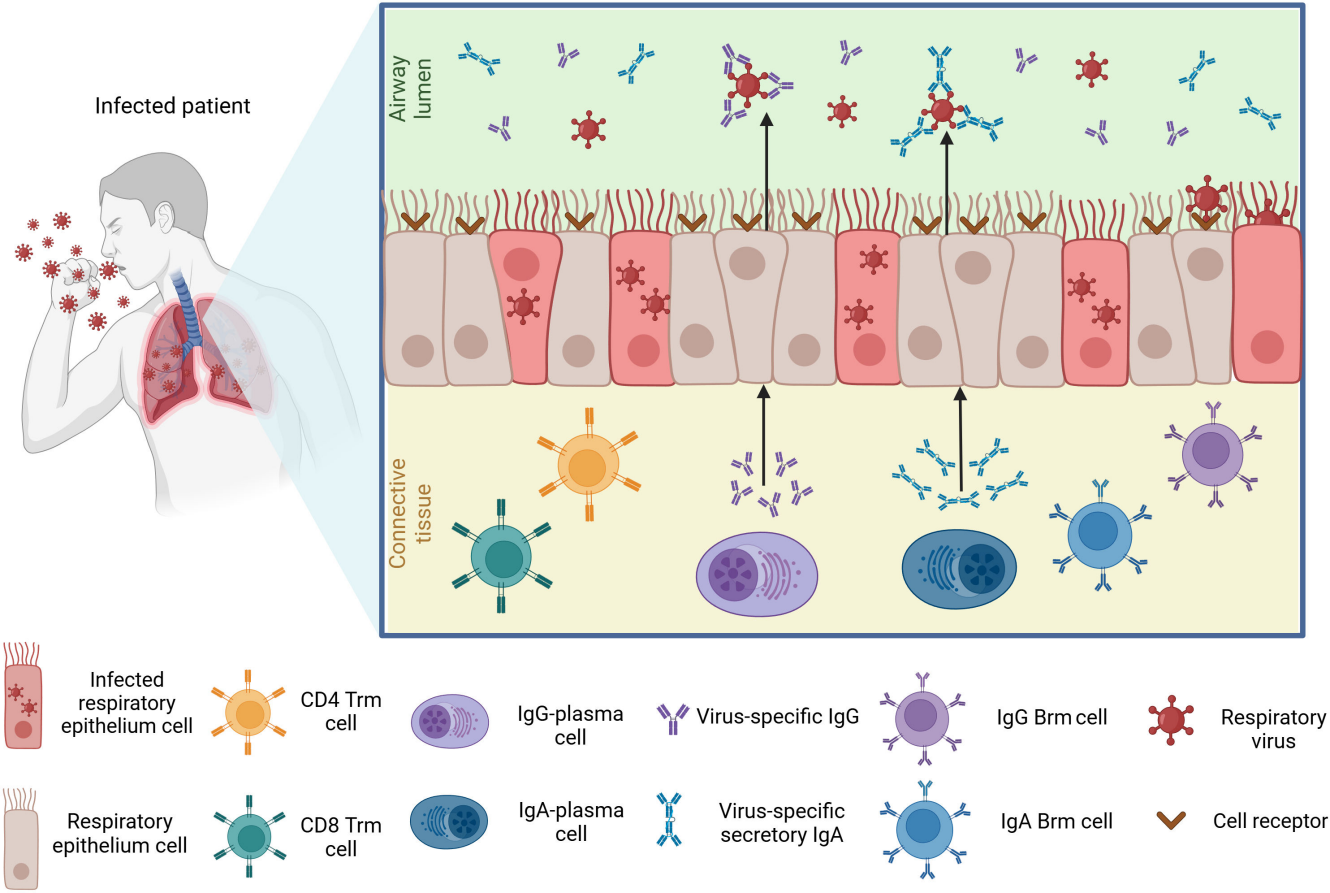
Mucosal immune components in the respiratory tract following viral infection. After a primary infection, epithelial cells lining the airway lumen become infected, which then initiates an immune response. The mucosal immune system is characterized by tissue-resident memory T cells (CD4^+^ and CD8^+^ T_rm_), which reside in the connective tissue and contribute to immune activation upon reinfection. B cell-derived plasma cells produce virus-specific immunoglobulins, with IgG-plasma cells secreting IgG and IgA-plasma cells generating secretory IgA (S-IgA), which is transported across the epithelium into the airway lumen to neutralize free virus particles and prevent infection. Memory B cells (B_rm_), including IgG^+^ and IgA^+^ B_rm_ subsets, are established in the respiratory mucosa and persist in the tissue to provide long-term immunity. Upon re-exposure to the same respiratory virus, these B_rm_ cells rapidly differentiate into plasma cells that secrete virus-specific IgG and S-IgA antibodies. Figure created with BioRender.com.

Respiratory viral infection induces local cytokine and chemokine release that provides innate defense, recruits specific leukocyte populations, and drives adaptive immune responses. In local lymphoid tissues or draining lymph nodes, these signals promote CD8^+^ and CD4^+^ T-cell differentiation, B-cell antibody production (including virus-specific mucosal secretory IgA and IgG), and the formation of tissue-resident memory T (T_rm_) and B (B_rm_) cells that protect against reinfection (85–88) (Fig. 1).

#### T lymphocytes

Antigen presentation and stimulation by APCs in the local milieu or draining mediastinal lymph nodes in the lung initiates T cell activation, clonal expansion, and differentiation into a heterogeneous pool of effector (T_eff_) and long-lived memory T cells. In viral respiratory diseases, severe outcomes, such as cytokine storms, are often linked to delayed or impaired T-cell responses and viral clearance (76, 78, 89–91). In influenza patients, higher numbers of IFN-γ-producing T cells correlate with lower levels of serum cytokines and chemokines, whereas severe disease is marked by elevated IL-6, IL-8, MIP1α, MIP1β, and CCL2, underscoring the importance of a timely, effective T cell response (92). In severe COVID-19, these changes are accompanied by significant increases in innate lymphocyte populations, including γδT cells, NK cells, and mucosal-associated invariant T (MAIT) cells (91, 93). Patients also show higher IL-6, IL-8, and CCL2 (94) in the respiratory tract lumen than in the blood, as well as increased proportions of neutrophils, intermediate CD14^+^ CD16^+^ monocytes, activated HLA-DR^+^ CD38^+^ CD4^+^ T cells, effector memory-like CD4^+^ and CD8^+^ T cells (93), and clonally expanded T_rm_17 cells (94), reflecting this highly inflammatory local environment.

#### Tissue-resident memory T cells

In contrast to circulating systemic memory T cells, long-lived T_rm_ cells reside in the respiratory mucosa near sites of infection, distinguishable from central memory T cells (T_cm_) and effector memory (T_em_) by their CD8^+^, CD103^+^, CD69^+^, and CCR7^-^ phenotype. They initially arrive among the many T cells migrating to the infected respiratory mucosa to clear the virus as a unique KLRG1^−^ CD8^+^ T-cell precursor population that differentiates into T_rm_ under a hybrid transcription program that has commonalities with T_em_ and T_cm_. The transcription factors, *Hobit, Blimp1,* and *Runx3* downregulate genes required for T cell egress from non-lymphoid tissues, including *Ccr7*, *Klf2*, and *S1pr1* (95, 96), thereby retaining these cells locally. In addition, *Runx3* induces CD69 expression (96), which combines with S1PR1, the receptor for sphingosine 1-phosphate (97), internalizing it to block promigratory signaling. Locally produced TGFβ and IL-15 induce CD103 expression and promote T_rm_ longevity (98), respectively. These T_rm_ cells function as tissue sentinels and are rapidly activated upon reinfection, likely by nearby “standby” antigen-presenting cells and are critical for effective early secondary adaptive responses (99–101). Indeed, influenza-specific lung-resident CD4^+^ T_rm_ cells promote viral clearance and survival, whereas splenic memory CD4^+^ T cells fail to protect against lethal reinfection (102). Similarly, CD8^+^ T_rm_ cells produce IFN-γ more rapidly than systemic CD8^+^ T_em_ cells and provide antigen-specific protection against influenza and Sendai viruses in the lung; their presence is also associated with reduced pro-inflammatory mediators, including CXCL1, CCL2, IL-6, and TNF (103). Innate lymphocytes, such as NKT and NK cells, that differentiate under transcription programs (95) similar to T_rm_ are also present in previously influenza-infected lung parenchyma and likely promote T_rm_ activation on reinfection (46). T_rm_ cells are also found in the lungs post-SARS-CoV-2 infection (104–106); however, they are not induced in the lung tissues by peripherally administered SARS-CoV-2 mRNA vaccines, which primarily elicit systemic memory T- and B-cell responses (106–108).

Collectively, these findings highlight the rapid and effective antiviral protection mediated by T_rm_. Thus, while SARS-CoV-2-specific lung T_rm_ remain to be fully evaluated for efficacy (108, 109), current data suggest that they may provide more persistent protective immunity, especially when the virus is prevalent in the community and re-infection is likely (110, 111).

#### B lymphocytes

In the lung, primary defense is largely mediated by IgA, the production of which exceeds the combined output of all other antibody isotypes. Two subclasses exist, IgA1 and IgA2. IgA2 is less susceptible to bacterial proteases due to its shorter hinge region and accounts for ~30% of IgA in bronchoalveolar lavage (BAL) fluid. In the lung mucosa, B cells produce IgA as a dimer linked by a J chain. Dimeric IgA binds the polymeric Ig receptor (pIgR) on the basolateral surface of epithelial cells and is transcytosed to the airway lumen, where cleavage of pIgR releases secretory IgA (S-IgA). The relative hydrophilicity of S-IgA enables ready incorporation into respiratory tract mucus. As a dimer, having four antigen-interactive arms substantially increases its avidity for invasive pathogens, potentially aggregating them and blocking epithelial attachment. In contrast, serum IgA is predominantly monomeric IgA1 produced in the bone marrow, with limited access to mucosal sites (112–114).

In mice, primary influenza infection induces markedly higher IgA and IgG levels in the BAL than in serum after intranasal compared to parenteral inoculation (115). Numbers of B cells producing IgA in the lungs of mice vaccinated with the adenovirus-vectored vaccine, ChAdOx1 nCoV-19, were also much higher using parenteral prime/intranasal boost than a parenteral-only prime/boost protocol (116). The humoral response to SARS-CoV-2 infection follows a similar pattern. In naïve patients, antibody-secreting cells expand transiently in the blood, while circulating follicular helper T (Tfh) cells increase over weeks, coinciding with rising serum IgG, IgM, and IgA. IgA levels decline during convalescence, but IgG and IgM maintain levels similar to the acute phase. Receptor-binding domain (RBD)-specific IgG seroconversion reaches 100% by convalescence, while IgM and IgA seroconversion remain at ~50%–76% throughout the acute and convalescent stages. Spike- and RBD-specific antibody titers correlate with neutralization, with maximal activity observed when multiple isotypes are present, suggesting complementary and/or synergistic effects (91). In COVID-19 patients, the respiratory mucosa contains high levels of IgG and IgM, with the RBD specificity of antibodies correlating with SARS-CoV-2 neutralization capacity, while serum is enriched for FcγR-binding IgG antibodies (93, 117). In severe disease, detectable SARS-CoV-2-specific anti-RBD IgM and IgG, and to a lesser extent IgA, in saliva correlate with serum levels (93, 118, 119). This is potentially a simpler approach to measurement and evaluation of anti-SARS-CoV-2 responses, but mechanistically, since very little systemic IgA evidently transudes or is actively transported across the mucosa from the blood (120, 121), it implicates the oropharynx and potentially the salivary glands, rather than the blood, as the principal origin of IgA antibodies detected in saliva. This indicates that SARS-CoV-2 infection mobilizes contiguous mucosal immune defenses in the oro-nasopharyngeal cavities and the respiratory tract. Indeed, URT sampling from infected individuals contains SARS-CoV-2-specific IgA^+^ and IgG^+^ memory B cells, CD8^+^ and CD4^+^ T_rm_, and B_rm_ (122).

Primary infection generates substantial numbers of B_rm_ in the respiratory tract. In influenza infection, B_rm_ persists over the long term in the iBALT along the LRT and is also distributed throughout the alveoli. They express CXCR3 and share features with T_rm_, e.g., expression of low levels of CCR7, CD62L, and S1PR1, as well as expression of CD69, but not CD103 (88, 123). Upon rechallenge, alveolar B_rm_ rapidly mobilize and home within 24 h to early sites of infection in the LRT, differentiate into plasma cells, and expand approximately threefold. This process is initiated by AMs, which sense incoming virus and promote IFN-γ production by NK and CD8^+^ T cells. IFN-γ induces neutrophil secretion of the CXCR3 ligands, CXCL9 and CXCL10, driving B_rm_ recruitment. Interestingly, the proportion of these newly formed plasma cells producing virus-specific IgG increases approximately fourfold, at the expense of those producing IgM, while proportions of IgA-producing cells remain similar (46). The rapid kinetics of this local recall response reduce early viral burden, limiting host cell infection and systemic spread, thereby decreasing disease severity and transmission. This temporal advantage underpins effective immunity following natural infection or vaccination.

While systemic immunity induced by natural respiratory infection and parenteral vaccination is important against re-challenge, especially if the virus spreads systemically, the induced local response in natural infection remaining after pathogen clearance at both innate and adaptive levels clearly confers a substantial temporal advantage critical to the efficacy of the response to any future challenge. The importance of this proximity is also emphasized by systemic studies in which vaccine re-challenge in the ipsilateral arm resulted in better and more rapid recall antibody responses than re-challenge in the contralateral arm (80).

## INTRANASAL VERSUS PARENTERAL VACCINES

**Fig 2:**
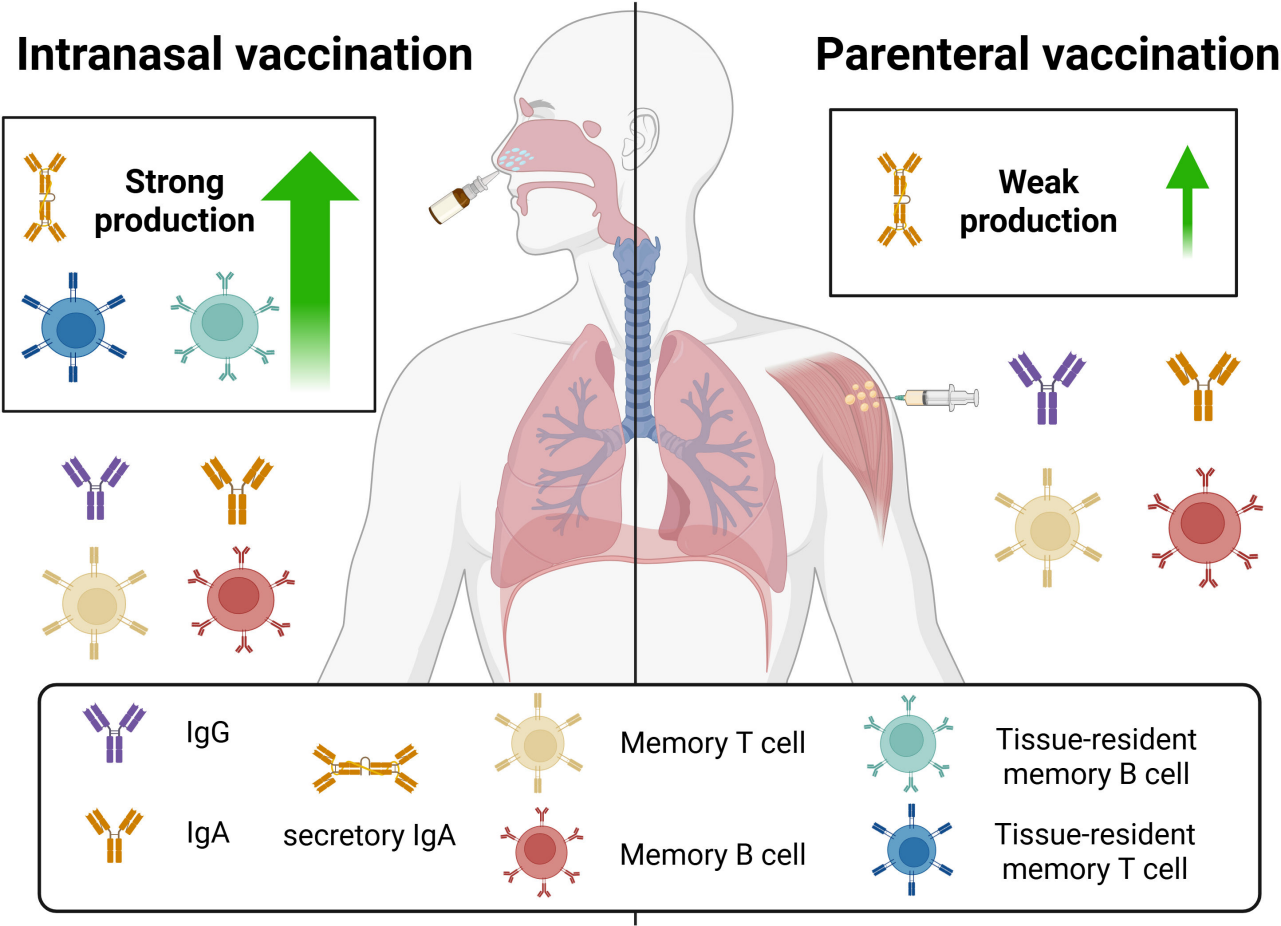
Comparison of immune responses induced by intranasal and parenteral vaccination. Intranasal vaccination elicits strong mucosal immunity, including high levels of S-IgA and IgG and the establishment of tissue-resident memory T and B cells in the respiratory tract. In contrast, parenteral vaccination mainly induces systemic immunity with limited mucosal antibody responses and does not generate tissue-resident memory cells. S-IgA at mucosal sites is dimeric, whereas serum IgA is monomeric. Figure created with BioRender.com.

**Fig 3:**
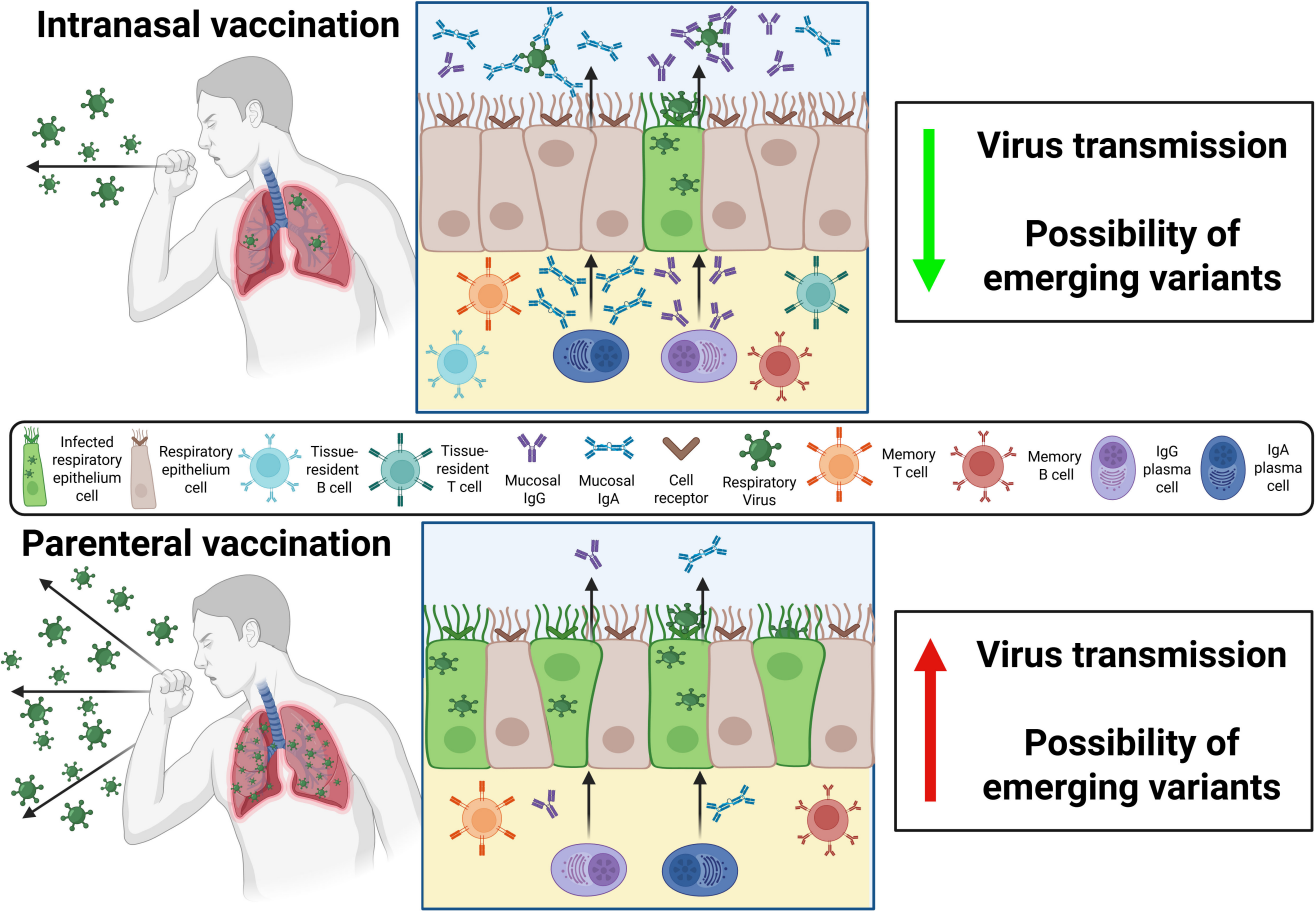
Comparison of immune protection induced by intranasal and parenteral vaccination upon respiratory virus exposure. Intranasal vaccination stimulates strong mucosal immunity. This localized response reduces viral infectivity and enhances early viral clearance, thereby decreasing virus shedding, transmission, and the likelihood of new variant emergence. In contrast, parenteral vaccination elicits primarily systemic immunity (e.g., serum IgG), which provides weaker mucosal protection, allowing increased viral infectivity and replication in the respiratory epithelium and consequently raising the risk of virus shedding, transmission, and variant emergence. Figure created with BioRender.com.

Mucosal vaccines are defined by correlates of protection centered on mucosal S-IgA and T_rm_ and B_rm_ responses, distinct from the systemic immunity elicited by parenteral vaccination (Fig. 2). Intranasal vaccination can reduce community viral transmission and may therefore lower the risk of new variant emergence, compared to parenteral vaccination (Fig. 3) (107). Preclinical studies with intranasal ChAdOx1 nCoV-19/AZD1222 showed reduced viral shedding and protection against SARS-CoV-2 variants, despite the vaccine’s original approval for intramuscular use (124–126). FluMist, a licensed influenza nasal spray, induces humoral and mucosal responses, including IgG, local IgA, and antigen-specific T cells (127, 128). Similarly, a recent intranasal SARS-CoV-2 vaccine was reported to stimulate robust mucosal and systemic neutralizing antibody and T-cell responses (129). In contrast, parenteral vaccination, while it may produce transient levels of S-IgA, does not stimulate differentiation of T_rm_ or B_rm_ (130). A study comparing subcutaneous and intranasal immunization reported higher serum IgA levels and more IgA^+^ antibody-secreting cells in bone marrow after intranasal vaccination, although higher doses were required to match IgG responses from subcutaneous delivery (131). Another study found that children vaccinated intramuscularly with mRNA vaccines had only modest salivary IgA1 responses, unlike the strong IgA2 responses observed after natural SARS-CoV-2 infection (132) (Fig. 2). This inability of parenteral vaccines to induce mucosal IgA was also evident in a cohort study following a fourth SARS-CoV-2 dose (133). However, a different study showed systemic and mucosal production of spike-specific IgG and IgA antibodies after intramuscular administration of the mRNA vaccines Spikevax and Comirnaty (134).

## MUCOSAL VACCINE ADJUVANTS

Not all vaccines require adjuvants; for example, live attenuated vaccines are capable of stimulating immune responses comparable to wild-type infection. However, adjuvants are often used to enhance the efficacy of mucosal vaccines across different platforms. Selecting an appropriate adjuvant is critical, as it can preserve antigen bioavailability, improve delivery, engage innate immune receptors, modulate mucosal barriers and secretions, induce mucosal cellular responses, and recruit or activate immune cells (Table 2) (135–144). Adjuvants can also provide a dose-sparing effect, as shown in multiple parenteral vaccine studies, without reducing efficacy (145–147). Different mucosal adjuvants have been evaluated in vaccination against respiratory viruses. While most clinically approved lipid nanoparticle (LNP) formulations have been developed for parenteral mRNA delivery, their ability to enhance antigen expression and stimulate innate immune pathways has generated interest in adapting these systems for mucosal administration. Notably, lipid nanoparticle (LNP) formulations, initially developed for parenteral mRNA delivery, are used in the approved mRNA vaccines mRNA-1273 (Moderna) and BNT162b2 (Pfizer) (19, 148). However, the successful translation of parenteral mRNA platforms to mucosal delivery remains challenging, although such approaches are currently being explored.

**TABLE 2:** Challenges of mucosal vaccine and strategies to overcome them

Challenges of mucosal vaccine	Examples of strategies
Poor antigen bioavailability	Incorporate protective delivery vehicles, such as nanoparticles (139–141), liposomes (149), and virus-like particles (150–152), to protect antigens from degradation and enhance stability
Inefficient antigen delivery and uptake	Enhance antigen uptake (33), using mucoadhesive agents (153, 154), penetration enhancers (143, 155), and optimization of the vaccination route and formulation (156, 157)
Mucosal barrier and secretions	Modulating mucosal site using nanoparticles and permeation enhancers (141, 158, 159)
Engage innate immune receptors	Employ pattern-recognition receptor agonists, such as TLR4 (33), CpG oligodeoxynucleotides (150, 160, 161), STING agonist (155), and monophosphoryl lipid A (162)
Induce mucosal immune response	Use prime-boost vaccination strategies (163) and design multivalent antigens (152, 153, 164, 165)
Recruit and activate immune cells	Targets immune cells such as dendritic cells directly (135, 142, 166)

### SARS-CoV-2

Several mucosal SARS-CoV-2 vaccine candidates incorporate adjuvants to enhance local immune responses. CIGB-669 (Mambisa) is an intranasal RBD protein subunit vaccine using hepatitis B core antigen (HBcAg) as an adjuvant, which stimulates Toll-like receptor 2 (TLR2), TLR3, TLR7, TLR8, and TLR9 (167) and increases anti-RBD IgG and neutralizing antibodies against Beta, Delta, and Omicron variants (168). ACM-001, an intranasal S protein subunit vaccine encapsulated in artificial cell membrane (ACM) polymersomes with the TLR9 agonist CpG 7909, targets dendritic cells and induces robust serum IgG, neutralization, and cross-reactivity against Delta and Omicron in hamsters (160, 161, 169). A different intranasal subunit vaccine, NanoSTING-SN, a liposomal spike-nucleocapsid subunit vaccine formulated with an endogenous STING agonist, elicits durable serum IgG and cross-reactive responses against Omicron, SARS-CoV, and MERS-CoV, supporting its potential as a pan-sarbecovirus vaccine (164). Also, the Th1/Th17-polarizing adjuvant Bordetella colonization factor A enhances long-lasting neutralizing antibodies and T_rm_ when used as a booster (170). Recently, a novel intranasal heteromultimeric S and N fusion protein subunit vaccine complexed with a mucoadhesive carrier, Nc, made with maltodextrin, effectively stimulated humoral and cellular immunity, with particularly elevated IgA levels in nasal and BAL secretions (153). The lack of translation of mRNA vaccines from parenteral to mucosal administration is mainly due to challenges in mRNA uptake in the mucosa and rapid degradation of nucleic acid (171). Various materials to deliver mRNA vaccines safely have been studied (148, 172–174). Jeong et al. successfully administered an ovalbumin mRNA vaccine intranasally using a photoactivated cationic polymer. Application of laser light enhanced the vaccine escape from endosomes, increased mRNA expression at the cellular level, and stimulated translocation of mRNA into the cytoplasm (175). This approach induced splenic CD4^+^ and CD8^+^ T-cell responses and IFN-γ production in mice.

### Influenza virus

Currently, there is no approved mucosal vaccine for influenza virus that uses an adjuvant. In efforts to identify effective mucosal adjuvants, Alum, MPL (TLR4 agonist), and CpG DNA (TLR9 agonist) have been shown to expand long-lived IgG- and IgA-producing cells and increase antibody levels in serum and mucosal tissues (162). Mannatide, a novel adjuvant, outperformed MF59 in an inactivated trivalent influenza vaccine administered through the intranasal route and enhanced HI titers against H1N1 and H3N2, antigen-specific serum IgG and IgA, mucosal S-IgA, splenic lymphocyte proliferation, and IFN-γ production (176). Furthermore, a mucoadhesive CS-IONzyme formulation also improved intranasal influenza vaccine efficacy in mice, increasing protection from 30% to 100% against lethal influenza A challenge (154). Flagellin from the *Vibrio vulnificus* or *Salmonella typhimurium* is another potent adjuvant that enhances systemic and mucosal antibody responses in influenza subunit vaccines (177, 178).

### RSV

As with influenza, adjuvanted mucosal RSV vaccines are currently in clinical trials. A nano-emulsion (NE)-adjuvanted RSV F protein subunit vaccine generated strong systemic and mucosal antibody responses without airway hyperreactivity, avoiding undesirable Th2-biased immunity when administered intranasally (179). NE has been shown to aid antigen uptake in nasal epithelial cells, activate TLR2 and TLR4 pathways, and induce heterogeneous cytokine production in Th1, Th2, and Th17 cells (166, 180). In a challenge study using cotton rats, RSV prefusion protein vaccine formulated with the endogenous STING agonist 2′3′-cGAMP (cyclic guanosine adenosine monophosphate) inhibits viral replication in the nose and lungs, while in mice it induces high S-IgA levels and Th1 responses after two doses (170). A poly-sorbitol transporter (PST) nanoparticle adjuvant combined with recombinant RSV A2 fusion protein enhances respiratory tract antigen uptake; however, although RSV-PST elicits robust systemic and mucosal antibody responses, it fails to generate functional antigen-specific CD8^+^ T_rm_ in the lung parenchyma (171).

## DELIVERY OF MUCOSAL VACCINES

Today, intranasal vaccination is limited to respiratory viruses like influenza and SARS-CoV-2 (130, 181). Other mucosal delivery routes, including sublingual, oral, and pulmonary, have also been explored for respiratory viruses, depending on the pathogen and target mucosal tissue (Table 3) (156, 182, 183). Structural, physiological, and immunological differences across these sites necessitate tailored strategies and delivery systems for effective vaccination. Robust mucosal immunity in the URT is especially important for preventing disease progression and blocking SARS-CoV-2 transmission (157, 184, 185). To achieve this, vaccines must overcome epithelial barriers and reach NALT and BALT, enabling antigen trafficking to cervical and mediastinal lymph nodes and stimulating strong local immune responses (186). Innovative delivery approaches, such as microneedle and microjet technologies, have shown promise in eliciting both systemic and mucosal immunity during oral vaccination (187, 188).

**TABLE 3:** Mucosal vaccination routes and immunization sites for respiratory viruses

Vaccination route	Delivery methods	Site producing immune response	Viruses
Intranasal	Intranasal spray, intranasal droplets	URT and LRT	SARS-CoV-2 (129, 189–191), influenza virus (192–194), RSV (195–198), hMPV (199, 200)
Pulmonary	Dry powder inhaler, aerosolizer, endotracheal delivery	URT and LRT (focuses are mainly on the LRT)	SARS-CoV-2 (201–203), RSV (204)
Oral	Tablet, liquid	Lungs and gut	SARS-CoV-2 (205, 206), influenza virus (158, 207, 208), RSV (209)
Sublingual	Biodegradable sublingual dissolving microneedle, sublingual droplets	Sublingual and lungs	SARS-CoV-2 (210, 211), influenza virus (183, 212, 213)
Buccal	Mucoadhesive film	Buccal and lungs	SARS-CoV-2 (214)

### Intranasal administration

One of the most widely studied approaches for respiratory vaccine delivery is intranasal administration using sprays or drops. These are easy to administer and target the nasal mucosa, primarily the URT, to induce mucosal immunity (192, 215, 216). In the development of second-generation COVID-19 vaccines, numerous studies have shown that intranasal vaccination induces both systemic and mucosal immune responses, elicits T-cell responses, and provides cross-reactive protection against multiple variants (129, 189–191). Similar findings have been reported for other viruses, including influenza (192–194), RSV (195–198), and hMPV (199, 200).

### Pulmonary administration

Aerosolized inhalation, delivered via nebulizers or aerosolizers, is an alternative mucosal vaccination route that targets the lower respiratory tract (LRT) and enhances immune responses (217, 218). Compared with intranasal delivery, pulmonary vaccination induces stronger humoral and cellular immunity, with reported cross-reactivity against multiple SARS-CoV-2 variants (201–203, 219). In an RSV vaccination study comparing intranasal and intrapulmonary routes, intrapulmonary vaccination induced more durable neutralizing antibody responses, reduced RSV replication, and improved clinical outcomes (204). Notably, targeting the LRT has also shown benefits for non-respiratory viruses, including human papillomavirus and hepatitis B (151) (220).

### Oral administration

Oral vaccines offer several advantages, including ease of administration and induction of gut mucosal immunity, while also eliciting lung immune responses that may limit SARS-CoV-2 transmission (221). Delivery of influenza vaccine to mesenteric lymph nodes induces mucosal S-IgA in nasal, lung, and vaginal secretions (207). Two SARS-CoV-2 oral vaccines based on the adenovirus type 5 platform, VXA-CoV-1 (205) and r-Ad-S (206), demonstrated robust mucosal immunity in the presence of S-IgA that is cross-reactive against other variants. Similar protective mucosal immunity has also been reported for influenza viruses (158, 207, 208) and RSV (209).

### Sublingual administration

The sublingual route is another promising oral mucosal site, enriched in APCs and lymphoid tissue capable of inducing both systemic and mucosal immunity (222, 223). Compared with intranasal or pulmonary delivery, sublingual vaccination may reduce the risk of Bell’s palsy and limit direct antigen exposure to vital organs (224, 225) (226). A novel technology, a biodegradable sublingual dissolving microneedle delivering SARS-CoV-2 spike protein, induces S-IgA secretion, reduces lung pathology, and lowers pro-inflammatory cytokines and chemokines (210). Yamamoto et al. reported that Poly(I:C)-adjuvanted RBD vaccines administered sublingually elicit robust immune responses in macaques (211), while a similar influenza HA vaccine induces mucosal and systemic immunity without inflammatory cytokine induction (183). In a subsequent follow-up study comparing intranasal administration of the same vaccine candidate, sublingual administration was associated with fewer adverse effects in the brain, lungs, tongue, and submandibular lymph nodes in mice and macaques (227). Sublingual influenza vaccination consistently protects mice from lethal viral challenge (212, 213).

### Buccal administration

Buccal vaccination is an underexplored approach but can elicit robust systemic and mucosal immunity (228). Using mucoadhesive films delivering a SARS-CoV-2 mRNA/LNP vaccine, Esih et al. demonstrated systemic immune responses comparable to intramuscular vaccination, together with induction of mucosal S-IgA (214).

In general, compared with parenteral vaccination, needle-free mucosal vaccination improves safety for vaccinators and recipients, eliminates needlestick injuries, and reduces community transmission risk (229). Needle-free vaccination is also likely to reduce non-compliance among needle-phobic individuals (230, 231), requires less healthcare training compared to syringe and needle vaccination, and is faster and safer (229).

## CURRENT MUCOSAL VACCINES ON THE MARKET AND IN DEVELOPMENT

### mRNA vaccines

Advances in mRNA vaccine technology have attracted global attention, particularly since the onset of the COVID-19 pandemic. In contrast, most mucosal vaccines currently approved or in development still rely on traditional platforms, such as live-attenuated, whole-cell inactivated, or viral vectors (Table 4). The limited translation of mRNA vaccines from parenteral to mucosal administration is primarily due to challenges associated with efficient mRNA uptake at mucosal surfaces, rapid nucleic acid degradation, and the toxicity of lipid nanoparticles, which remains a major hurdle for mucosal mRNA vaccine delivery. For SARS-CoV-2, Baldeon Vaca et al. recently developed an intranasal mRNA-LNP vaccine that induced robust systemic spike-specific binding (IgG and IgA) and neutralizing antibodies, though spike-specific mucosal IgA was undetectable. Interestingly, while overall T-cell populations did not change across treatment groups, a specific increase in CD4^+^ T cells was observed following intranasal vaccination (191). Evidence for influenza is more limited: Yahyaei et al*.* reported their mRNA vaccine induced both systemic and mucosal antibody responses, along with antigen-specific IFN-γ secretion (192), whereas another study found that intramuscular or intranasal vaccination of mRNA-HA induced weak or no immune responses (232).

**TABLE 4:** Approved respiratory virus mucosal vaccines and those in clinical trials

Target virus	Vaccine platform	Vaccine name	Background	Status	Reference
SARS-CoV-2	Viral vector vaccine	iNCOVACC	Chimpanzee adenoviral vector	Approved	(189)
		Convidecia	Adenovirus type 5 vector	Approved	(233)
		MPV/S-2P	Murine pneumonia virus vector	Clinical trial	(234)
		B/HPIV3/S-6P	Bovine/human parainfluenza virus vector	Clinical trial	(235)
		Pneucolin	Influenza virus vector	Approved	(236)
		SC-Ad6-1	Adenovirus vector type 6	Clinical trial	(237)
		COV2	Adenovirus vector type 5	Clinical trial	(238)
		AZD1222	Simian adenovirus vector	Clinical trial	(239)
	Subunit vaccine	Mambisa or CIGB-669	RBD protein with Hepatitis B virus core antigen (HBcAg) as adjuvant	Clinical trial	(168)
		ACM-001	S protein encapsulated in ACM polymersomes with CpG 7909	Clinical trial	(169)
		LVT-001	Trimeric fusion protein consisting of S and N proteins fused to Fc domain	Clinical trial	(240, 241)
Influenza virus subtypes A and B	Live attenuated virus vaccine	FluMist or Fluenz	Cold-adapted, temperature-sensitive, trivalent live attenuated influenza virus (2 type A, H1N1 and H3N2, and 1 type B)	Approved but replaced by quadrivalent or Fluenz Tetra	(165)
		Nasovac-S	Cold-adapted, trivalent live attenuated influenza virus (2 type A, H1N1 and H3N2, and 1 type B, Victoria lineage)	Approved	(242)
		FluMist quadrivalent or Fluenz Tetra	Cold-adapted, temperature-sensitive, quadrivalent live attenuated influenza virus type (2 type A, H1N1 and H3N2, and 2 type B, Yamagata lineage and Victoria lineage)	Approved	(243)
Influenza virus subtype A		Pandemic influenza vaccine H5N1 AstraZeneca	Cold-adapted, temperature-sensitive, live attenuated influenza virus type A, H5N1	Approved	(244)
Respiratory syncytial virus	Live attenuated virus vaccine	CodaVax-RSV	Codon-deoptimization	Clinical trial	(245)
		RSVT	Not disclosed	Clinical trial	(246)
		RSV ΔNS2 Δ1313/1314L	NS2 gene-deletion and temperature-sensitivity mutation in the polymerase gene	Clinical trial	(247)
		LID/ΔM2-2/1030s	Deletion of RSV ribonucleic acid synthesis regulatory protein M2-2 and genetically stabilized temperature-sensitivity mutation 1030s in the RSV polymerase protein	Clinical trial	(248)
		RSV 6120/∆NS1 and RSV 6120/F1/G2/∆NS1	NS1 gene modification	Clinical trial	(249, 250)
	Viral vector vaccine	BLB-201	Parainfluenza virus type 5 encoding RSV F protein	Clinical trial	(251, 252)
Human metapneumovirus		B/HPIV3/hMPV-PreF-A and B/HPIV3/hMPV-F-B365	Not disclosed	Clinical trial	(253)

### Subunit vaccines

Subunit mucosal vaccines share similar challenges with mRNA vaccines, as without suitable carriers or adjuvants they cannot effectively cross the mucosal layer to deliver antigens to immune cells. For SARS-CoV-2, RBD-based subunit vaccines induced robust mucosal immunity and protected animals against variants (254–258). As a heterologous booster, the XBB.1.5 RBD vaccine elicited long-term immunity characterized by stimulation of T_rm_ cells, germinal centers, and memory B cells in the respiratory tract (257). S protein subunit vaccines also showed protection (259–261); notably, Mao et al*.* demonstrated that even an unadjuvanted S subunit vaccine conferred robust cellular and humoral immunity in the mucosa (261). Leekha et al. developed a potential pan-sarbecovirus vaccine using a multi-antigen (spike and nucleocapsid) subunit combined with an endogenous STING (stimulator of interferon genes) agonist (NanoSTING-SN), which induced broad immunity against sarbecoviruses (164). Similar progress has been made for influenza, where various subunit formulations, including adjuvants (159, 262–264) and VLPs (152, 265), protected animals from diverse variants and subtypes. Other novel methods, such as targeting the neonatal Fc receptor (FcRn) to enhance S-IgA, have also shown promise (255, 266). In the case of RSV, multiple adjuvanted subunit vaccines elicited systemic, humoral, and cellular immunity (267, 268). However, a G protein-based subunit vaccine exacerbates lung pathology via IL-13 and mucin hypersecretion, indicating a Th2-skewed immune response (269). In contrast, a phosphatidylcholine-liposome vaccine irradiated with low-energy electrons (PC-LEEI-RSV) reduced viral load after challenge and induced neutralizing antibodies in mice (270)*.*

### Viral vector vaccines

Viral vectors have the added advantage in mucosal vaccine development of naturally penetrating the mucosal layer and stimulating adaptive immunity without adjuvants. For SARS-CoV-2, two intranasal adenoviral vaccines, iNCOVACC and Convidecia, are approved and show cross-protection against recent variants (189, 233). iNCOVACC, a chimpanzee adenoviral vectored intranasal vaccine, induced stronger serum neutralizing antibody responses and higher mucosal IgA levels with up to fivefold greater salivary IgA than the intramuscular vaccine Covaxin, while generating comparable T cell immunity and cross-neutralizing activity against Omicron BA.5 in seronegative participants (189). Similarly, Convidecia, an adenovirus type 5 (Ad5)-based inhalable booster, elicited higher neutralizing antibody levels when given orally versus intramuscularly (233). Other candidates are also progressing, such as MPV/S-2P, a murine pneumonia virus vector that expresses a prefusion-stabilized SARS-CoV-2 spike protein (S-2P) and is now in phase 1 trials (NCT06441968) (234), which in macaques provided complete airway protection after boosting by inducing systemic and mucosal anti-spike antibodies and airway CD4^+^/CD8^+^ tissue-resident memory T cells (271). NIAID is also funding a safety study (NCT06026514) of a nasal spray vaccine candidate, B/HPIV3/S-6P (bovine/human parainfluenza vector), expressing prefusion-stabilized spike protein (235). In 2024, dNS1-RBD (Pneucolin), a live-attenuated influenza vector encoding the RBD, received Emergency Use Authorization for adults ≥18 years (236). Another promising approach is NDV-HXP-S, a Newcastle disease virus vector that induces strong RBD-specific antibodies and is undergoing phase 1 testing (NCT05181709) to compare intranasal and intramuscular administration (272, 273).

Beyond SARS-CoV-2, adenoviral vectors remain widely used for mucosal influenza vaccines, inducing immunity across multiple variants (232, 274). Liu et al*.* generated an H5N1 vaccine using their DelNS1 LAIV system (Deleted-NS1 Live Attenuated Influenza Viral Vector Vaccine system) that offered robust neutralizing antibody, S-IgA, and T-cell responses in mice (193). Similar strategies are being applied to RSV, where adenoviral (275, 276), Sendai (277), and NDV vectors (278) have been explored. Notably, PanAd3-RSV and MVA-RSV, based on chimpanzee adenovirus and modified vaccinia virus Ankara, respectively, showed good safety and induced strong humoral and cellular immunity in adults (275, 279–281).

### Live attenuated virus vaccines

Live attenuated virus vaccines have historically been highly effective in preventing systemic infections, such as smallpox, influenza, measles, mumps, and rubella (282). Modern approaches use codon deoptimization, codon-pair deoptimization, or targeted mutations in viral genomes of SARS-CoV-2 and other pathogens (129, 283–286), preserving native antigen conformations, and inducing broad immune responses while minimizing risk of reversion to pathogenic forms (287–289). For SARS-CoV-2, several groups have developed intranasal live-attenuated vaccines that elicit both systemic and mucosal immunity, comparable to mRNA vaccines, with Adler et al. and Liu et al. reporting strong IgG and IgA responses, induction of T_rm_ and B_rm_, cross-variant protection, and robust CD4^+^ and CD8^+^ T-cell responses against S, N, and M viral structural proteins (129, 190). Similar strategies have long been applied to influenza, where the first approved intranasal vaccine, T/LAIV (FluMist or Fluenz), a live attenuated tetravalent vaccine, protects individuals aged 2–49 by inducing antigen-specific T cell responses, particularly CD4^+^ memory T cells (165). T/LAIV was later replaced by Q/LAIV (Fluenz Tetra or FluMist Quadrivalent) to broaden coverage of circulating B strains. Although early versions showed limited protection against H1N1, efficacy was improved through vaccine strain updates (243, 290–292). Other LAIVs, such as India’s Nasovac-S, have shown modest protection (293–296), while AstraZeneca’s monovalent H5N1 LAIV demonstrated HA-specific CD4^+^ T-cell priming, supporting pre-pandemic immunization efforts (242, 244, 297). Progress has also been made for RSV, where the bivalent Metavac vaccine displaying RSV and hMPV F proteins protected mice by inducing neutralizing antibodies with strong affinity for both RSV and hMPV (199). Clinical development includes Codagenix’s CodaVax-RSV in phase 1 trials (NCT04919109) (298), several NIH-funded studies of recombinant live-attenuated RSV vaccines (NCT01893554, NCT03596801, and NCT04520659) (247–250, 299, 300), and Sanofi’s RSV vaccine candidate, RSVT, in multiple trials (NCT05687279, NCT06252285, NCT06397768), and showing high serum neutralization in infants and toddlers (246, 301–303). Finally, for hMPV, an NIAID-funded phase 1 study (NCT06546423) is evaluating a live attenuated Human Parainfluenza Virus 3 (HPIV3) vector expressing the hMPV fusion glycoprotein in HPIV3-seropositive children (253).

#### Heterologous prime-boost strategy

Unlike homologous prime-boost vaccination, which uses the same vaccine, heterologous prime-boost vaccination combines vaccines from different platforms or delivery routes that express the same or overlapping antigens (304). The goal is to integrate complementary immune effects to elicit broader humoral, cellular, and sometimes mucosal protection while limiting reactogenicity (163). This approach gained attention during the COVID-19 pandemic when mixed schedules were adopted to overcome vaccine supply issues or reduce adverse effects (305). For SARS-CoV-2, heterologous regimens combining mucosal and parenteral vaccines have been more effective than homologous ones. In a phase 3 trial, intranasal iNCOVACC boosted adults primed with Covaxin or Covishield (AstraZeneca), enhancing T-cell memory and increasing IgG- and IgA-secreting plasma cells, with fewer local adverse events (306). Similarly, Lapuente et al. demonstrated that an intranasal Ad5 vector booster following BNT162b2 priming fully protected mice, inducing mucosal IgA, T_rm_ in the lungs, and cross-variant neutralization (307), while Shamseldin et al. showed that a mucosal booster after intramuscular spike protein vaccination sustained neutralizing antibodies and T_rm_ for 3 months in mice (170). Furthermore, Xing et al. developed a dual-specificity chimpanzee adenovirus vector (AdC-HATRBD) targeting both SARS-CoV-2 and influenza, which enhanced immunity when combined with the licensed vaccine ZF2001 and quadrivalent inactivated influenza vaccine (308). Studies of influenza vaccines have shown that an intramuscular H3 mRNA prime, followed by an intranasal H3 protein subunit boost, produced the strongest cross-reactive antibodies and mucosal immunity across influenza strains (309), while a bivalent subunit vaccine encoding influenza N and M1 proteins plus RSV F protein, delivered by intramuscular prime and intranasal boost, induced protection against influenza A H3N2 or H1N1, as well as RSV A, with potent Th1-skewed responses and high lung CD8^+^ T_rm_ (310). Extending this prime-boost strategy to RSV, Yang et al. reported that priming mice with HBsAg DNA intramuscularly and boosting intranasally with liposome-encapsulated HBsAg protein enhanced mucosal and systemic immunity (149). In contrast to intranasal booster-based regimens, Blanco et al*.* showed that intranasal priming with adjuvanted RSV F protein, followed by intradermal boosting in cotton rats, reduced RSV replication without exacerbating lung pathology (162).

### Conclusion

Mucosal vaccines offer a powerful approach to protecting against respiratory viruses by inducing both local and systemic immunity at the site of virus entry. Their development against pathogens, such as SARS-CoV-2, influenza, RSV, and hMPV, is crucial for pandemic preparedness. With diverse delivery methods and platforms under active investigation and strong global support, research is poised to accelerate the development of more effective mucosal vaccines.
